# The Use of Big Data *via* 5G to Alleviate Symptoms of Acute Stress Disorder Caused by Quarantine Measures

**DOI:** 10.3389/fpsyg.2021.569024

**Published:** 2022-02-23

**Authors:** Hossein Hassani, Nadejda Komendantova, Stephan Unger, Fatemeh Ghodsi

**Affiliations:** ^1^Research Institute for Energy Management and Planning, University of Tehran, Tehran, Iran; ^2^Advancing Systems Analysis Program, International Institute for Applied Systems Analysis (IIASA), Laxenburg, Austria; ^3^Department of Economics & Business, Saint Anselm College, Manchester, NH, United States; ^4^Qazvin University of Medical Sciences, Qazvin, Iran

**Keywords:** big data, psychological stress, quarantine, COVID-19, mental health, 5G

## Abstract

This article investigates the role of Big Data in situations of psychological stress such as during the recent pandemic caused by the COVID-19 health crisis. Quarantine measures, which are necessary to mitigate pandemic risk, are causing severe stress symptoms to the human body including mental health. We highlight the most common impact factors and the uncertainty connected with COVID-19, quarantine measures, and the role of Big Data, namely, how Big Data can help alleviate or mitigate these effects by comparing the *status quo* of current technology capabilities with the potential effects of an increase of digitalization on mental health. We find that, while Big Data helps in the pre-assessment of potentially endangered persons, it also proves to be an efficient tool in alleviating the negative psychological effects of quarantine. We find evidence of the positive effects of Big Data on human health conditions by assessing the effect of internet use on mental health in 173 countries. We found positive effects in 110 countries with 90 significant results. However, increased use of digital media and exclusive exposure to digital connectivity causes negative long-term effects such as a decline in social empathy, which creates a form of psychological isolation, causing symptoms of acute stress disorder.

## Introduction

During the ongoing COVID-19 pandemic many countries have introduced measures to manage and mitigate the risk of the virus spreading. People have been exposed to conditions of severe uncertainty in relation to case fatality rates, the spread of the virus, and the costs of risk mitigation measures. The impact of this uncertainty is connected to morbidity, mortality, and risk, and has resulted in increased levels of panic and anxiety across almost all societies.

Previous disease outbreaks such as SARS or Ebola have shown that anxiety can appear in a community as the result of many factors such as death and the number of new cases reported frequently by media (Rubin and Wessely, 2020). However, the recent COVID-19 outbreak caused drastic measures to be taken by governments globally, e.g., imposing stay-at-home orders. These dramatic measures increased the level of anxiety because they interfered with the natural desire and necessity of people to socially interact with other people. Generally, quarantine implies a lack of control and a sense of being trapped, which is intensified when families are isolated (Rubin and Wessely, 2020). There is sufficient evidence that quarantine can cause substantial psychological, emotional, and financial challenges. To be more effective, quarantine requires the person at risk to be isolated and follow proper infection-control steps within the quarantine area (Hawryluck et al., 2004). Moreover, self-isolation can lead to severe symptoms of acute stress disorder (ASD) (Brooks et al., 2020).

The impact of quarantine measures was studied extensively in relation to pandemics such as SARS or Ebola. Various interviews and surveys among those who experienced the quarantine measures revealed that the impact on mental and psychological health can be enormous including isolation and separation, post-traumatic stress, depression, avoidance behavior, irritation, sadness, annoyance, anxiety, frustration, guilt, helplessness, loneliness, and nervousness. The quarantine measures can also lead to symptoms of ASD, which is characterized by panic and anxiety, and stigmatization, even following social isolation. The major difference of the current situation in comparison to the SARS or Ebola pandemics is the role of social media. Through social media various fake news, rumors and misinformation was spread which further increased the negative impact and the levels of anxiety. This is also defined as digital stress, caused by a huge variety of communication channels and contradictory information. The COVID-19 crisis showed a correlation between psychological consequences and the utilization of digital media.

Big Data is a comparatively new discipline of data science that investigates how massive data sets can be interpreted to harvest significant insights and information. It is worth mentioning that traditional data processing methods/approaches are not suitable for gathering, warehousing, and the analysis of big data. The five V’s of big data (velocity, volume, value, variety, and veracity) are the five primary and innate characteristics of Big Data (for more information, see, for example, Hassani et al. (2019a,b, 2020, 2021).

So-called “Big Data” have significant potentials for the health sector as it can help monitor the spread of disease. They can also help to increase the quality of medical services. Big Data can also help to create transparent information for people, and this helps to alleviate some of the most negative impacts on mental health such as anxiety and uncertainty, which leads to additional stress.

The deployment of Big Data will benefit from the introduction of 5G technology, which will connect more than 50 billion people and will allow the analysis of a much larger percentage of digital data than before. The 5G network also enables the transmission of Big Data in real time, including up-to-date diagnostics. The health sector welcomes the deployment of 5G, as this technology will benefit from the features of high-speed networks by reducing the time required to analyze large amounts of data.

Influenced by mobility restrictions, people in quarantine use digital channels of communication much more frequently to compensate for the restricted mobility. During the pandemic, this resulted in a greater volume of Big Data being generated during quarantine. This is on the one hand a chance for further data analysis, but on the other, personal data protection, and the appropriate storage of data should be considered.

The usage of the Big Data approach in health systems is still minimal. This paper discusses the sustainable use of Big Data with the application of 5G technology, concerning data protection issues. Such usage can be beneficial in the health sector and could help to address the negative impacts on mental health that have arisen after quarantine measures. The paper discusses the benefits of 5G technology, such as increased speeds of data transmission and how they can be beneficial for the health sector and calls for further research in this area to understand all consequences of the implementation of 5G technology.

## Common Symptoms Caused by Quarantine

Quarantine is critical for managing infectious diseases, and may be successful from an epidemiological perspective to control exposure to infection. However, it has some side effects. It has a dramatic effect on the finances, emotions, and psychology of the people who have had to stay at home completely (Brooks et al., 2020). A review of related articles shows that psychological stress is one of the critical negative consequences of quarantine. Emotional disturbance, depression, low mood, irritability, and insomnia have been seen repetitively (Hawryluck et al., 2004; Brooks et al., 2020). It has been confirmed that the increased duration of quarantine is associated with growing post-traumatic stress disorder (PTSD) and ASD. PTSD and ASD are anxiety disorders with long-term psychiatric morbidity. From a psychological point of view, PTSD is developed by someone who experienced a life-threatening event. It can be recognized by some criteria, such as increased vigilance and heart beating (Bryant, 2017). Some evidence indicates that post-traumatic stress can increase significantly in more than a 10-day quarantine. Moreover, ASD is identified by PTSD symptoms, which occur early after trauma. It could be a short-term side effect of staying completely at home. ASD can lead to anxiety, insomnia, poor concentration, and exhaustion. As a result of this problem, the number of people who changed careers, relocated for work, and/or considered resigning has increased (Brooks et al., 2020). In one study, 87.4 percent of people who stayed at home reported psychological and corporeal distress after the outbreak (Lee et al., 2005).

Fear is a common emotion experienced by most people in quarantine, fear for their health, or fear of infecting others. People quarantined often experienced a lack of communication, even with their family members. As a result, depression and low mood have been reported in some studies. One qualified study about the psychological effect of SARS quarantine in Toronto showed that symptoms of depression were observed in about 31.2 percent and in this study, the presence of depressive symptoms was highly correlated with PTSD symptoms (Hawryluck et al., 2004). In the December 2019 outbreak, most of the countries around the world applied quarantine once again for reducing the spread of the disease managing better of the COVID-19 pandemic. According to WHO technical guidance notes about COVID-19 “the main psychological impact to date is elevated rates of stress or anxiety, as new measures and impacts are introduced (especially quarantine and its effects on many people’s usual activities, routines or livelihoods) levels of loneliness, depression, harmful alcohol, and drug use, and self-harm or suicidal behavior are also expected to rise” (CDC Covid-19 Response Team, 2020). Some other articles provide scientific evidence about similar negative consequences caused by quarantine. These articles outline a tremendous increase in frustration, boredom, and distress reactions such as rage and rigid fear of getting illness even in those not exposed (Caleo et al., 2018) and lasting impact on health-risk behaviors, for instance, overuse of tobacco and alcohol. With increased numbers of people experiencing social isolation, people may experience stigmatization (Pan et al., 2005; Ćosić et al., 2020).

A significant number of scientific works exist about the impact of quarantine on mental and psychological health. These studies focused on the impacts of Ebola, SARS, and other diseases. There were also various studies on the impact of quarantine, mainly because of SARS, on the mental health of inhabitants in different countries such as Taiwan (Bai et al., 2004), China (Mihashi et al., 2009), Hong Kong (Lee et al., 2005), and South Korea (Jeong et al., 2016), but also the United States (Sprang and Silman, 2013), Canada (Blendon et al., 2004), and Australia (Braunack-Mayer et al., 2013). Many studies have identified the impact of quarantine measures on mental health, because of the Ebola pandemic in Liberia (Pellecchia et al., 2015) and Sierra Leone (Caleo et al., 2018).

Many of these studies examine the impact of quarantine measures on various social groups. For instance, a study that compared post-traumatic stress symptoms among parents and children found that children who were in quarantine had four times more frequent post-traumatic stress symptoms than children who were not in quarantine. A significant share of parents (28%) showed symptoms of trauma-related mental health disorders in comparison to a less significant (6%) share of parents who were not in quarantine (Sprang and Silman, 2013). Another study identified the impact of quarantine measures on hospital workers. It found that a significant share of workers showed symptoms of depression even 3 years after the event. In total, 60% of workers who showed depressive symptoms also experienced quarantine measures (Liu et al., 2012). Another study found that people who were quarantined because they were in close contact with people who had infectious diseases experienced fear (20%), nervousness (18%), sadness (18%), and guilt (10%). Positive feelings such as happiness (5%) or feelings of relief (4%) were reported in fewer studies (Reynolds et al., 2008).

Only a few studies have conducted correlation analysis of the impact of quarantine measures on mental health dependent on demographic variables such as marital status, age, education, living with other adults, or having children. However, no significant evidence of the correlations between these variables was found (Hawryluck et al., 2004). Two variables had a significant influence on mental health, such as professional occupation and previous psychological sicknesses (Jeong et al., 2016). For instance, among those who have been quarantined, health workers have more severe symptoms of post-traumatic stress syndrome than the general public. Furthermore, quarantined workers feel more anger, annoyance, fear, frustration, guilt, helplessness, isolation, loneliness, and nervousness. They are also more likely to think that they have an infection and that they will infect others (Robertson et al., 2004).

A number of studies have identified correlations between a negative impact on mental health and factors such as the duration of quarantine, fear of infection, frustration and boredom, and inadequate supply and information. Longer durations of quarantine resulted in more frequent post-traumatic syndromes, avoidance behavior, and anger (Marjanovic et al., 2007). The negative impacts of quarantine were significantly higher if the duration of the measures was longer than 10 days (Hawryluck et al., 2004). Some studies found correlations between fears and factors such as having young children or expecting children or being physically vulnerable physically (Desclaux et al., 2017). There was also a correlation between how strongly the daily routine was affected, the reduction of social contact, and feelings of boredom and frustration (Blendon et al., 2004). The inadequate supply during quarantine, such as the delivery of medical items, food, water, or groceries, was strongly connected with feelings of anxiety and anger, which were present even several months after the release of quarantine measures (Wilken et al., 2017). Inadequate, confusing, or contradictory information about the pandemic or necessary actions was cited as one of the main stress factors (Braunack-Mayer et al., 2013). Often the various messages, differences in style, approaches, and content used by various public institutions were perceived as stress factors (DiGiovanni et al., 2004). Lack of clarity about the risk and its seriousness significantly influenced risk perceptions (Desclaux et al., 2017). Perceived lack of transparency from public authorities about the severity of pandemics also influenced risk perceptions (Braunack-Mayer et al., 2013). Lack of clear guidelines and perceived difficulties in coping with quarantine measures contributed significantly to the development of post-traumatic stress symptoms (Reynolds et al., 2008).

The main difference to the situation of today is that the role of social media during the earlier Ebola and other pandemics was not as significant. Social media make the spread of information much faster and almost universal as almost everybody has access to social media. Together with various kinds of messages and news, social media are also spreading misinformation or in some cases even disinformation. Existing widespread information, a great number of rumors, fake news, and conspiracy stories in conditions of high uncertainty and high impact on human life is creating further negative impacts on mental and psychological health, in addition to the already stressful situation. This situation also adds to the effects described above, including anxiety and various psychological disorders. Negative perceptions of quarantine measures can also be influenced by social media and are connected with factors such as perceived loss of freedom, uncertainty over disease status, and boredom. These factors can lead to feelings including irritation or depression or even to dramatic consequences such as suicide.

## How Big Data Can Help Alleviating Stress Symptoms

We are now living in an era of “Big Data” marked by significant growth and an exponential rate of data generation. This fact creates a huge opportunity for public health in finding possible patterns and trends across populations and possibly foretelling disease outbreaks based on powerful search engine results combined with a huge data warehouse and analytics. For instance, using API information, and geographic location to identify disease transmission, combined with other auxiliary information such as analyzing posts on Twitter could lead to promising results (Rosen et al., 2020). By analyzing people’s data in quarantine, we can recognize the main problems, and possibly find a solution. Improving the level of public health is a vital aim for all countries, so by using data, we can achieve this aim. People in quarantine have used digital media more than usual. They have tried to catch more information about their worries. Lack of education and having false information leads to increased psychological problems.

An important factor is the availability of a standard instrument. For instance, based on collected data, this could be a survey that reflects the full spectrum of psychological aspects of quarantine and useful approaches to overcoming many related issues. Such an instrument would be extremely useful for possible outbreaks in the future. A standardized instrument facilitates a connection and probable correlation between the psychological responses to outbreaks for various infectious causes and also could be utilized to control symptoms over time (Hawryluck et al., 2004). Categorizing these data would provide a guideline for managing public health problems as well as alleviating stress symptoms during the quarantine. It should be noted that although employing the Big Data approach for public health intervention is still very minimal, with growing data convergence, impressive analytic power, and the broad range of applications and its success, one expects Big Data and smart techniques to become one of the increasingly common prime choices (Rosen et al., 2020).

Belle et al. (2015) indicated two devices that are available everywhere and continuously generated data and information; telemetry and physiological signal monitoring devices. However, data and information generated from these devices have not been deposited, as would be expected during this age of technology. Nevertheless, there have been some efforts toward collecting, depositing, employing, and analyzing telemetry and continuous physiological extracted signals and data from monitoring to improving patient care and management (see, for example, Mackenzie et al., 2008; Apiletti et al., 2009; Bodo et al., 2013; Hu et al., 2014).

The computational aspect of Big Data aids the health care system and organizations across the globe to have real-time decision-making. This enables them to enhance and advance the quality of healthcare services as well as cost reduction (Ta et al., 2016).

It is important to consider the compatibility of front-end as well as back-end systems. While certain environments run on different operating systems, the devices that gather the data and send it to be stored (i.e., on clouds) might run on different systems. This incompatibility might cause delays or inefficiencies in the transmission process. It might also lead to misleading analytics or even system crashes.

There are various applications of Big Data and its related techniques for tackling fundamental issues and trends in healthcare analytics research, with various applications ranging from text mining and predictive modeling to pattern and face recognition (Sun and Reddy, 2013).

One psychological factor of how Big Data can help alleviate stress symptoms during a pandemic is through the creation of transparent information for affected people. A very significant factor adding to stress is uncertainty. During a pandemic, quarantined people have a lot of time due to the imposed restrictions. However, this suddenly gained time might turn into stress when people are confronted with an indefinite course of the measures taken by authorities, as well as contradictions in the health statistics published. The data generated through surveillance tracing, health statistics, and reports can help people to alleviate these kinds of stress symptoms, enabling them to stay informed.

As well as creating more accurate information on the current status of the pandemic, Big Data can create better predictions about the future of the outbreak (Bansal et al., 2016).

To test how Big Data can help to improve health conditions we first want to give an overview of the existing literature in this area. As a proxy for Big Data, we relate general internet usage to the generation and use of Big Data. It, therefore, makes sense to take a closer look at health studies conducted since the emergence of the internet, since this time frame covers a regime change that potentially reveals changes in health-related conditions. An obvious way in which Big Data and the internet were and continue to be used was to look up potential diagnoses and treatments for certain health problems. Trotter and Morgan (2008) study internet usage in 2000 and 2006 for health related information. Through a survey, they found a significant increase in looking up health related information on the internet.

Several studies have been conducted to investigate the introduction of 5G and data provision. A recent study by Rizatto (2020) estimates that 5G users on average consume up to 2.7 times more mobile data compared to 4G users. The study comprised six countries: South Korea, United Kingdom, Japan, United States, Australia, and Germany, and found an average consumption of 15GB of mobile data for 5G users. By estimating the effect of regular internet usage, which is assumed to be mainly based on 4G, on mental health, we can therefore deduct the potential effect of the introduction of 5G on improving health conditions.

A white paper by Cisco (2020) indicates that nearly two-thirds of the global population will have internet access by 2023. They also outline that over 70 percent of the global population will have mobile connectivity by 2023 and that 5G devices and these connections will account for over 10 percent of global mobile devices and connections by 2023. Another study by Cisco (2016) found that more than 500 billion Internet of Things (IoT) devices, from sensors to actuators, to medical devices, will be connected to the internet by 2030.

In a survey, Pew Research Center (2018) found that 95 percent of Americans own a cell phone and 77 percent have smartphones, whereas, for low-income segments of the population, including those in African-American and Hispanic communities, wireless connectivity is most likely their only online access (Turner Lee, 2019).

Turner (2019) also mentions the importance of 5G for health care services. This is because 5G and IoT are broadly applied to life-saving devices and other applications in health care where it is “*imperative that they operate as anticipated, without fail, every time*.” Lee further mentions significant examples of the importance of the 5G applications for health such as home health sensing, which is a critical intervention for chronic disease patients that uses microphones in smartphones to replicate spirometers, which measure the airflow in and out of lungs for patients with chronic obstructive pulmonary disease (COPD). The data collected is used by doctors to monitor the disease’s progression in patients in real-time (Turner, 2019).

Another important application of 5G, and internet usage, is among pregnant women seeking health information. Javanmardi et al. (2018) conducted a review study of 16 articles and found that the use of the internet by pregnant women was driven by information needs, ease, and speed of access, and finding people with the same situation. The article shows the importance of accessibility and speed provided by 5G.

Ducrot et al. (2021) analyze and describe the evolution of internet use as a source of health information between 2010 and 2017 and found that the use of the internet as a source of health information rose between 2010 and 2014 (from 37.3 to 67.9%, *P* < 0.001) but decreased significantly in 2017 (60.3%, *P* < 0.001). They outline that the reason for the recent decrease in internet usage was the parallel decrease in trust in the quality and reliability of information found online. The authors stress the need for public health authorities to increase citizens’ eHealth literacy and to provide alternative trustworthy sources combining the popularity and accessibility of general health information websites. Their results highlight the importance of 5G to enable patients a quicker verification of health information found online.

## Data and Potential Analysis

To show the potential of 5G to alleviate stress symptoms, and thus, to improve mental health conditions we tested the impact of global internet usage in each country on its population health condition. For this purpose, we took telecommunication network usage as a proxy and individuals using the internet in % of the population (Worldbank, 2021) and for the measurement of mental health the Disability-Adjusted Life Years (DALYs), which considers not only the mortality associated with a disorder but also years lived with disability or health burden (Ourworldindata, 2021).

Our sample size comprises 173 countries for which we found available data from Worldbank. We took yearly data from 1990 to 2019 for each country, *k*, and regress the annual changes of DALYs on the percentage of the population of country *k* using the internet, *INT*,


(1) $$D\,A\,L\,Y_{k}\,\left(t\right)=\alpha_{k}+\beta_{k}\,I\,N\,T_{k}\,\left(t\right)+\in_{t},$$

for all countries, *k* = 1,..,173. We assume that, as discussed above, broader usage of the internet leads to an alleviation effect of stress symptoms, causing an improvement of mental health in the long-run.

By estimating these parameters we are able to deduct what impact the average increase in internet usage will have on the mental health of a population.

## Results

The results show that a significant amount of countries exhibit a negative beta, which means that an increase in internet usage improves mental health conditions. For most of the countries, we found a significant reduction of a population’s share of the total disease burden. In detail, as reported in Supplementary material Appendix, out of 173 countries we found that 90 have significant results (*P* < 0.10), 73 countries highly significant (*P* < 0.05), and 110 countries had a negative beta, meaning that 63.58% of the analyzed countries show a positive impact of increased internet usage on mental health.

When looking at continents, we classify the countries into 6 regions: North America, South America, Arabia, Asia/South Pacific, Europe, and Africa. When analyzing the relative share of countries with a negative beta to the total number of countries in the region, and the level of significance, we found the following results.

Table 1 provides an overview of the relative amount of countries that exhibit a negative beta, compared to the total number of countries in that region. We can see that North America leads by far in terms of the positive effect of internet usage on mental health, followed by South America, the Arabic region, Asia/South Pacific, Europe, and Africa. The reason for Africa coming last might be under-developed internet access and infrastructure, respectively, and awareness of the population of the positive effect of internet usage on their health condition.

**TABLE 1:** Relative share of countries with a negative beta to the total number of countries in each region, ranked, including the relative amount of significant results in each region.

	Negative Beta			Significant (*P* < 0.05)	
1.	100%	North America	1.	63.64%	Arabia
2.	89.29%	South America	2.	53.85%	Africa
3.	81.82%	Arabia	3.	53.57%	South America
4.	78.95%	Asia/South Pacific	4.	50.00%	North America
5.	72.50%	Europe	5.	31.58%	Asia/South Pacific
6.	30.77%	Africa	6.	25.00%	Europe

The results also indicate the relative share of countries for which we found the negative beta to be significant (*P* < 0.05). We found that the Arabic region yielded the most significant results, followed by the African continent, South and North America, Asia/South Pacific, and finally Europe. The reason for Arabia and Africa having the majority of significant results might lie in the growth rates of internet usage in these countries. The average internet usage growth rate per year of 56% in Arabic countries, and the average yearly growth rate in Europe is less, at around 40%.

To illustrate these results we further analyzed 20 countries and found that 13 out of 20 showed significant results.

Table 2 shows the test results for the G20 countries, analyzing the impact of internet usage per population on the mental health condition of the corresponding country. Out of the 20 countries, we found that 17 countries had a negative beta. The only exceptions are Japan, Italy, and Russia, which exhibit a negative intercept, implying an already high degree of internet usage for these populations in general. However, the test results of these countries are not significant and exhibit a very low R^2^.

**TABLE 2:** Summary table of G20 DALY mental health condition of population regressed on internet usage per country population from 1990 to 2019.

	Intercept	Beta	*P*-value	*R* ^2^
Germany	0.0129273	−0.019027	4.21E–05***	0.495244
United States	0.0103237	−0.018925	0.00655***	0.260299
Australia	0.0108802	−0.014969	0.009383***	0.240627
Canada	0.0024749	−0.00637	0.085847*	0.113418
Saudi Arabia	0.0372408	−0.040256	5.75E–07***	0.721396
India	0.0235893	−0.005949	0.80903	0.002482
Russia	−0.013848	0.0489164	0.093026*	0.108727
South Africa	−0.024372	0.1929178	9.29E–06***	0.551068
Turkey	0.0362545	−0.059203	0.003692**	0.312225
Argentina	0.0094602	−0.007786	0.314633	0.042102
Brazil	0.0299709	−0.062219	4.68E–09***	0.752717
Mexico	0.0242918	−0.065685	2.94E–05***	0.50913
France	0.0042269	−0.002129	0.588921	0.011846
Italy	−0.000274	0.007386	0.336082	0.037054
United Kingdom	0.0109992	−0.009687	0.025947**	0.183165
China	0.0277782	−0.069235	1.17E–08***	0.763534
Indonesia	0.019823	−0.02437	0.770124	0.003963
Japan	−0.00697	0.0039311	0.464696	0.021577
South Korea	0.0157814	−0.011671	0.000771***	0.369465
EU	0.006929	−0.00761	0.023468**	0.188941

Significant codes: 0 – ***0.001, **0.05, *0.10; 1.

The test result shows that an extension of the internet and data transmission capacity, i.e., through the upgrade from 4G to 5G, has the potential to improve the mental health conditions of the populations in the countries where better accessibility and faster broadband is being established. This article will next discuss the potential role of 5G in a mental health crisis in detail.

## The Potential Role of 5G in a Mental Health Crisis

In telecommunication terminology, 5G is defined as the fifth-generation technology standard for cellular networks and is considered as the next generation of 4G networks (which currently provide connectivity to most cellphones). One of the notable advantages of 5G networks is that they provide greater bandwidth and faster download speeds.

It is worth mentioning that 5G is not just an extension of 3G and 4G, rather, it involves a mixed network combining 4G, Wi-Fi, millimeter-wave, as well as many other wireless access technologies. It explores and derives a deeper aspect from the data/information/signal extracted by a significant number of devices by integrating cloud infrastructure, a virtualized network core, intelligent edge services, and a distributed computing model. In terms of frequency bands, 5G utilizes high-frequency bands around and higher than 60,000 MHz whilst the majority of current mobile (cellular) communications utilize below the 3.5 MHz range. Generally, four factors can be considered as main factors to distinguish 5G from its predecessors: connected devices, fast and intelligent networks, back-end services, and extremely low latency. As such enhanced mobile broadband, machine-to-machine communications, artificial intelligence, and advanced digital services of 5G are increasingly employed in the medical sector (West, 2016).

It is expected that the 5G network will be deployed to connect 50 billion devices and 212 billion sensors in 2020. This will include smartphones, tablets, smartwatches, cars, machinery, appliances, and remote monitoring devices, and many others (King, 2016). Accordingly, a huge amount of valuable data will be generated and provide a golden opportunity to extract useful information. It is expected that 5G as a connected ecosystem empowers us to use a more substantial percentage of digital data (35%) than previously (5%) (Davis, 2017).

During a quarantine, symptoms of ASD are likely to occur. It is characterized by anxiety and panic, particularly stigmatization and social isolation of confirmed cases, survivors, and relations, which may escalate into further negative psychological reactions, including adjustment disorder and depression (Kinsman, 2012). During the epidemic, rapid, integration can maximize effective management of the psychological crisis. Psychological crisis intervention should be dynamic, adapted to setting different stages of the epidemic, i.e., during and after the outbreak. During the outbreak, mental health professionals should actively participate in the overall intervention process in the disease, so that the mental health and psychosocial response can be mobilized in a timely fashion (Mohammed et al., 2015). With the support for remote psychological intervention provided by the development of internet technology, especially the widespread application of 4G or 5G networks and smartphones, the new intervention model was developed to handle the present COVID-19 public health event (Zhang et al., 2020). Nevertheless, some evidence suggests that there is a correlation between psychological consequences and the utilization of digital media. Digital stress, which is defined as stress caused by negative interactions in emails, texts, social media, chat rooms, and forums, is an essential intervening factor (Steele et al., 2020).

There is an indication that digital media increase the perception of our communities and activities (Table 3). The association between digital media and stress is indirect. The increase in digital media usage is undoubtedly related to alterations in the structure of humans’ life (Hampton et al., 2016).

**TABLE 3:** 4G download speed reduction during COVID-19 pandemic across Asia, Europe, the Middle East, Africa, South, and Central America, and North America on a weekly basis between the last week of January and the fourth week of March (January 27 to March 29), (Opensignal, 2020).

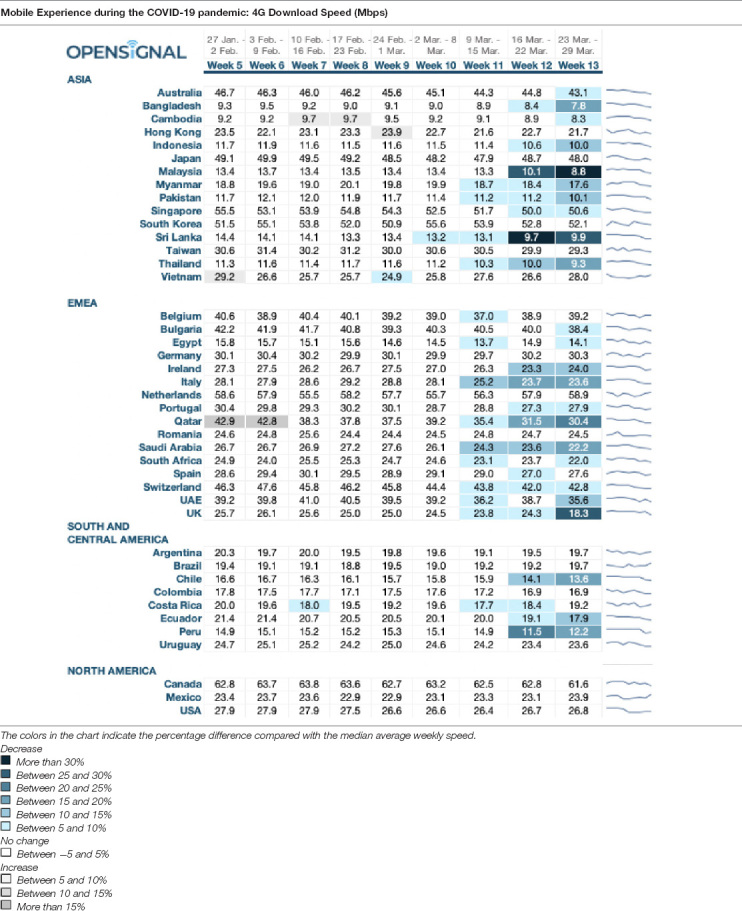

To analyze the potential role of 5G in a mental health crisis, one first needs to distinguish between the role of 5G in diagnostics and treatment. For instance, EMC in the context of 5G offers personalized emotion-aware services with the aid of mobile cloud computing and affective computing (Yu et al., 2015). As 5G allows to transmit Big Data in real time, real time diagnostics, as well as treatment, is possible. The speed factor is the fundamental reason that the 5G network could be successful in a mental health crisis. To date, cell phone applications have been used to collect data about patients that allow them to reach health professionals with just one text message. It is claimed that this system is to be effective as it represents a mobile crisis center (National Institute of Mental Health, 2020). The three times faster speeds of a 5G network provide a real time, no interruptions response to a patient’s need, which is what distinguishes this technology from the older ones and why it is beneficial. Data collection can reveal information such as location, movement, and whether the patient will receive immediate care.

Another benefit of the increased speed is that it will allow experts to reach patients that reside in rural areas, where other forms of connectivity are lacking. A survey revealed that 60% of rural citizens in the United States face shortages in access to mental health professionals (National Institute of Mental Health, 2020). The medical industry is also able to use the features of high-speed networks by greatly reducing the time required to read large amounts of data from patients, whether it is personal information, clinical research, or high-resolution MRI and CT imaging information.

5G also enables remote monitoring devices, such as wearable technology, to send patient health data to doctors instantly, while also being able to know what kind of space the patient is currently working through the sensor to achieve more comprehensive and adaptive sexual medical or nursing methods.

The interconnectivity of medical devices enables instant remote monitoring of the mental health of patients that show symptoms of ASD caused by quarantine measures. A reliable way to achieve direct monitoring would be by invasive deep brain stimulation (DBS) as is already performed by neurosurgeons to treat obsessive compulsive disorder, which is characterized by an obsessive behavior in which the patient cannot stop what they are doing, e.g., washing hands or cleaning their surroundings. It is well documented that by employing advanced technology, biomedical sensors can provide an impressive possibility to measure human physiologic parameters in a continuous, real-time, and non-intrusive manner (Seshadri et al., 2019).

In the case of Big Data, devices monitoring the mental health of quarantined people could be placed in or on the body to track the mental and physiological status, such as the emotional and vital functions of the person and send them in real time *via* the IoT to medical cloud centers. There, artificial intelligence backed systems could analyze data in real time and alert alarm medical response teams. They could even initiate emergency measures in cases where the condition of the person is deteriorating, which could lead e.g., to self-harming behavior. On the one hand, this means that immediate intervention is made possible through the utilization of 5G’s instantaneous connectivity. On the other, the long-term implications of quarantine measures could be analyzed using real-time data over a prolonged period of time, and thus reveal important insights to prevent symptoms of ASD.

These types of procedure would, of course, be highly questionable ethically but nevertheless, show the power and capabilities of 5G technology. Since neurostimulators are already widely used, e.g., to listen to and stimulate electric current in the brain at the same time, 5G could help to understand the symptoms of ASD caused by quarantine measures and even serve for instant diagnostics and treatment for vulnerable people (Manke, 2018). Combining the potential of 5G with the power of brain pacemakers or any other body-attached devices opens a huge potential for suicidal or depression treatment, or any other negative effects caused by long-term lockdowns.

We will next show the potential of 5G in medical applications by simulating the download speed of 5G compared to the 4G download speed (in Mbps) during the COVID-19 pandemic. The potential time saved indicates that the higher capacity enabled by 5G would allow medical response teams to react faster and thus save more lives when it comes to life-threatening mental conditions caused by symptoms of ASD caused by quarantine measures. It is important to note that the reduction in download speed took place in the absence of any real time health data monitoring, simply due to increased use of the internet. This means that in the presence of health data monitoring, the potential reduction in download speed, and transferrable data, would be even more significant. For our analysis we focused on the relation of data being not able to be transmitted due to reduction in download speed, compared to a 5G framework.

Table 2 provides data on the 4G download speed reduction across Asia, Europe, the Middle East, Africa, South, and Central America, and North America on a weekly basis between the last week of January and the fourth week of March (January 27 to March 29) (for more information regarding the data collection and source of the data, refer to https://www.opensignal.com).

Based on this data we compare a regular 5G transmission speed of 10 *giga*bits per second (Gbps). Regular data transmission channels *via* 4G face problems like Large Volume of Data, Bandwidth Fluctuations, and High Channel Error Rates. E.g., the amount of uncompressed data is large, even for a single patient. Transmitting a large amount of generated data over a wireless link in a real time fashion is a considerable challenge (Kang, 2014). Given a world population size of almost 7.79 billion globally, this means that a day worth of digital mental and physiological data would encompass 3.795 billion GB of data each day, or 43,923 GBps, additional on currently transmitted data.

Since our data set comprises 40 nations, totaling 62 billion people, a day worth of digital mental and physiological data would encompass 1, 31 billion GB of data each day, or 15,179 GBps. The total Mbps transferred over the 9 weeks across all 40 nations was 10,380 Mbps. Considering the average reduction over 9 weeks of −1.2% of data transfer speed across all 40 nations, this would result in an average loss of deliverable data of −0.28 Mbps/week, totaling −95.83 Mbps/week.

In total, this means that under the current 4G network capacities, the total pro-rata loss would be −0.92% or −140.15 Gbps across the 40 nations. Considering that a day of data potentially encompasses 500MB per person, the equivalent of a loss of −140.15 Gbps would be an uncovered amount of 500 people per second or 43.25 million people per day. Given the transmission speed of 10 Gbps, 5G would enable coverage of these 43.25 million people who might not be covered under the regular 4G network speed as these people might potentially need medical aid throughout a day due to ASD symptoms. The results of this study are in line with recent findings. For instance, Thomas et al. (2021) discuss “how” and how “effective” modern technologies are in controlling the COVID-19 outbreak. They also discussed the pros and cons of social media and television in ensuring global connectivity and awareness.

## Conclusion

Big Data can be useful for public health interventions, especially in crisis times like the COVID-19 pandemic. Even though quarantine is a critical method for managing infection and the spread of disease, it has side effects such as increasing the number of people who suffer from ASD and PTSD. A new intervention model that adopts digital media could alleviate this problem.

The development and rollout of the 5G network enables interconnectivity among devices at an incredible speed. While real time data about the physiological and emotional well-being of humans during a lockdown has not been generated yet, 5G opens the possibility of gathering data about not only the psychological status of quarantined people but also provides the possibility for instantaneous intervention by medical response teams in cases of deteriorating psychological and physiological conditions through real-time connection, e.g., AI-backed mental health monitoring systems. The IoT is therefore a useful tool for the 5G infrastructure to work on a comprehensive real-time health monitoring system that could benefit not only the affected people but also policymakers, enabling them to better understand the long-term health effects of quarantine and lockdown measures.

The present study took internet usage as a proxy for Big Data utilization and tested the effect of internet usage per population in 173 countries on the mental health conditions in the corresponding population, measured by the DALYs, which measures the years lived with disability or health burden. We found the positive effect of internet usage on mental health in 110 countries, out of 173, by looking at yearly changes in internet usage and the reduction in DALYs. The results show geographically interesting distributions as the effect seems to be biggest in North and South America, followed by the Arabic region, Asia and the South/Pacific, and then Europe and Africa. The distribution reveals certain conclusions, such as a higher awareness in populations in North and South America about the positive effect of using digital information on health conditions. Poor results in Europe indicate hesitation in the population about the digital information provided in improving health conditions, while the poor results in Africa might be caused by lack of infrastructure and accessibility to online sources as well as affordability.

Taking historical 4G download speed reductions during the COVID-19 pandemic, we calculate that given the transmission speed of 10 Gbps, 5G would enable the real time transmission of digital mental and physiological data of 43.25 million more people who suffer from ASD symptoms caused by quarantine measures per day to remote medical response teams, than under the current 4G capacities.

## Data Availability Statement

The raw data supporting the conclusions of this article will be made available by the authors, without undue reservation.

## Author Contributions

All authors listed have made a substantial, direct, and intellectual contribution to the work, and approved it for publication.

## Conflict of Interest

The authors declare that the research was conducted in the absence of any commercial or financial relationships that could be construed as a potential conflict of interest.

## Publisher’s Note

All claims expressed in this article are solely those of the authors and do not necessarily represent those of their affiliated organizations, or those of the publisher, the editors and the reviewers. Any product that may be evaluated in this article, or claim that may be made by its manufacturer, is not guaranteed or endorsed by the publisher.
