# Supplementary material for: The Use of Big Data via 5G to Alleviate Symptoms of Acute Stress Disorder Caused by Quarantine Measures
Source: Front Psychol. 2022 Feb 23;12:569024. doi: 10.3389/fpsyg.2021.569024 (PMC8905680; doi:10.3389/fpsyg.2021.569024)
Supplement: Supplementary file 1 [file Data_Sheet_1.docx]

**Appendix**

| **Country** | **Intercept** | | **P-Value** | | $\boldsymbol{R}^{\boldsymbol{2}}$ | | **Beta** | |  |
| --- | --- | --- | --- | --- | --- | --- | --- | --- | --- |
| Afghanistan | 0.00312028 | | 0.92790186 | | 0.00060588 | | 0.02461471 | |  |
| Albania | 0.22818565 | | 0.23981875 | | 0.06514961 | | -0.2552442 | |  |
| Algeria | 2.2507E-06 | | 0.0878826* | | 0.12663709 | | -0.3558611 | |  |
| American Samoa | 2.2507E-06 | | 0.0878826* | | 0.12663709 | | -0.3558611 | |  |
| Andorra | 0.26699563 | | 0.04862651** | | 0.18941357 | | -0.4352167 | |  |
| Angola | 9.8305E-08 | | 0.10518707 | | 0.12587931 | | 0.35479474 | |  |
| Antigua and Barbuda | 1.2574E-07 | | 4.0066E-06*** | | 0.66294013 | | -0.8142114 | |  |
| Argentina | 0.00227938 | | 0.31463267 | | 0.04210177 | | -0.2051872 | |  |
| Armenia | 0.00057272 | | 0.04202597** | | 0.17482772 | | -0.418124 | |  |
| Australia | 0.00360379 | | 0.00542746*** | | 0.30179632 | | -0.5493599 | |  |
| Austria | 0.00044166 | | 0.0271721** | | 0.18050628 | | -0.4248603 | |  |
| Azerbaijan | 0.00098822 | | 0.0807215* | | 0.14471708 | | -0.380417 | |  |
| Bahrain | 0.00026668 | | 0.00702815** | | 0.29817287 | | -0.5460521 | |  |
| Bangladesh | 2.2343E-15 | | 0.001886** | | 0.42357115 | | -0.6508234 | |  |
| Barbados | 0.00144977 | | 0.01010945 | | 0.27559254 | | -0.5249691 | |  |
| Belarus | 0.65574636 | | 0.13887269 | | 0.11159336 | | 0.33405592 | |  |
| Belgium | 0.00399644 | | 0.77850413 | | 0.00322318 | | 0.05677301 | |  |
| Belize | 0.38052239 | | 0.98309315 | | 2.4265E-05 | | 0.00492592 | |  |
| Benin | 5.9797E-16 | | 1.1544E-09 | | 0.84919048 | | 0.92151532 | |  |
| Bermuda | 0.01069354 | | 0.01367987** | | 0.2564136 | | -0.506373 | |  |
| Bhutan | 1.1337E-06 | | 0.25533963 | | 0.08002818 | | -0.2828925 | |  |
| Bolivia | 6.2833E-21 | | 2.9969E-11*** | | 0.88298704 | | -0.9396739 | |  |
| Bosnia and Herzegovina | 0.06613872 | | 0.08816202* | | 0.1384415 | | -0.3720773 | |  |
| Botswana | 0.9861749 | | 0.0101089** | | 0.27559596 | | 0.52497235 | |  |
| Brazil | 1.6315E-12 | | 4.6784E-09*** | | 0.75271704 | | -0.8675927 | |  |
| Bulgaria | 0.36115127 | | 0.55618838 | | 0.01527116 | | 0.12357652 | |  |
| Burkina Faso | 2.1367E-10 | | 0.16524693 | | 0.09397882 | | 0.30655966 | |  |
| Burundi | 0.00093495 | | 0.58392682 | | 0.01525783 | | -0.1235226 | |  |
| Cambodia | 1.5187E-13 | | 0.00028553*** | | 0.50841929 | | -0.7130353 | |  |
| Cameroon | 4.801E-05 | | 8.935E-06*** | | 0.65472072 | | 0.80914815 | |  |
| Canada | 0.28453208 | | 0.0858472* | | 0.11341814 | | -0.3367761 | |  |
| Central African Republic | 0.75273331 | | 0.03603571** | | 0.20166244 | | 0.44906841 | |  |
| Chad | 9.3226E-05 | | 0.00465993*** | | 0.35093935 | | 0.59240135 | |  |
| Chile | 0.00305873 | | 0.00506778*** | | 0.28393651 | | -0.5328569 | |  |
| China | 9.3138E-12 | | 1.1696E-08*** | | 0.76353362 | | -0.8738041 | |  |
| Colombia | 0.00367708 | | 0.20351825 | | 0.07241933 | | -0.2691084 | |  |
| Comoros | 6.7734E-05 | | 0.74032373 | | 0.00625441 | | -0.0790848 | |  |
| Costa Rica | 0.01274473 | | 0.01389471** | | 0.2268901 | | -0.4763298 | |  |
| Cote d'Ivoire | 5.7019E-08 | | 0.00031789*** | | 0.46809752 | | 0.68417653 | |  |
| Croatia | 0.39999616 | | 0.8134242 | | 0.00247192 | | -0.0497185 | |  |
| Cuba | 0.51173321 | | 0.03288335** | | 0.19898476 | | -0.4460771 | |  |
| Cyprus | 0.00974543 | | 0.72655597 | | 0.00518941 | | -0.0720376 | |  |
| Denmark | 0.58263875 | | 0.58023804 | | 0.01240325 | | 0.11136987 | |  |
| Djibouti | 1.7726E-10 | | 0.42010884 | | 0.03119932 | | 0.1766333 | |  |
| Dominica | 0.05863642 | | 0.00901468** | | 0.29486401 | | -0.5430138 | |  |
| Dominican Republic | 0.00018828 | | 0.00724706** | | 0.29628825 | | -0.5443237 | |  |
| Ecuador | 0.0012092 | | 0.9871376 | | 1.1548E-05 | | -0.0033982 | |  |
| El Salvador | 0.17898769 | | 0.68569919 | | 0.00836096 | | -0.0914383 | |  |
| Equatorial Guinea | 1.0905E-09 | | 0.04382267** | | 0.19704291 | | -0.4438952 | |  |
| Eritrea | 0.41087685 | | 0.93277381 | | 0.00049034 | | -0.0221435 | |  |
| Estonia | 0.7355537 | | 0.68830267 | | 0.00682303 | | 0.08260162 | |  |
| Eswatini | 1.4328E-06 | | 3.3764E-10*** | | 0.85285176 | | 0.92349973 | |  |
| Ethiopia | 2.0543E-08 | | 0.65318265 | | 0.00979789 | | 0.09898427 | |  |
| Finland | 0.03771269 | | 0.05884789* | | 0.13552252 | | -0.3681338 | |  |
| France | 0.0490822 | | 0.58892089 | | 0.01184625 | | -0.1088405 | |  |
| Gabon | 0.00976868 | | 1.4775E-05*** | | 0.63641898 | | 0.79775872 | |  |
| Georgia | 0.5797114 | | 0.22433077 | | 0.06945984 | | -0.2635524 | |  |
| Germany | 7.4642E-06 | | 4.2129E-05*** | | 0.49524389 | | -0.7037357 | |  |
| Ghana | 6.7885E-08 | | 0.00430042** | | 0.32782767 | | 0.57256237 | |  |
| Greece | 0.07022148 | | 0.00056565*** | | 0.38407743 | | -0.6197398 | |  |
| Greenland | 1.4027E-09 | | 6.7615E-07*** | | 0.68183433 | | -0.8257326 | |  |
| Grenada | 0.01687993 | | 0.00182297** | | 0.39210873 | | -0.6261859 | |  |
| Guam | 0.00038035 | | 0.00029737*** | | 0.45546076 | | -0.6748783 | |  |
| Guatemala | 4.8262E-08 | | 0.00027894*** | | 0.47439586 | | -0.688764 | |  |
| Guinea | 1.717E-11 | | 0.02061703** | | 0.22046908 | | 0.46954135 | |  |
| Guinea-Bissau | 0.00296962 | | 0.19304189 | | 0.08746495 | | 0.29574473 | |  |
| Guyana | 0.14726495 | | 0.54811909 | | 0.02455274 | | -0.1566931 | |  |
| Haiti | 0.85819192 | | 0.67331538 | | 0.00955834 | | 0.09776676 | |  |
| Honduras | 0.35766587 | | 0.8168019 | | 0.00261366 | | -0.051124 | |  |
| Hungary | 0.28729431 | | 0.66207927 | | 0.00776421 | | 0.08811474 | |  |
| Iceland | 0.0103837 | | 0.04626044** | | 0.1495993 | | -0.3867807 | |  |
| India | 2.3045E-09 | | 0.8090304 | | 0.00248178 | | -0.0498175 | |  |
| Indonesia | 0.05485326 | | 0.77012418 | | 0.00396257 | | -0.0629489 | |  |
| Iraq | 0.14687987 | | 0.87789866 | | 0.00162552 | | 0.04031771 | |  |
| Ireland | 0.00038777 | | 0.09881185* | | 0.10942783 | | -0.3307988 | |  |
| Israel | 0.12878696 | | 0.61693516 | | 0.01015795 | | -0.1007866 | |  |
| Italy | 0.92494912 | | 0.33608208 | | 0.03705393 | | 0.19249397 | |  |
| Jamaica | 0.22428465 | | 0.13857045 | | 0.09695942 | | -0.3113831 | |  |
| Japan | 0.03828678 | | 0.46469566 | | 0.02157727 | | 0.14689204 | |  |
| Jordan | 2.8685E-07 | | 0.76484404 | | 0.00435386 | | -0.0659837 | |  |
| Kazakhstan | 0.39911704 | | 0.06515981 | | 0.1462284 | | 0.38239823 | |  |
| Kenya | 0.02840486 | | 0.000621*** | | 0.43474989 | | 0.65935566 | |  |
| Kiribati | 7.6835E-12 | | 0.18715003 | | 0.09458292 | | 0.30754336 | |  |
| Kuwait | 0.70059473 | | 0.16442672 | | 0.0822937 | | 0.28686878 | |  |
| Latvia | 0.24302905 | | 0.50827882 | | 0.02218329 | | -0.1489406 | |  |
| Lebanon | 2.2264E-06 | | 0.00173165** | | 0.38006419 | | -0.6164935 | |  |
| Lesotho | 0.01854955 | | 0.00010977*** | | 0.53500277 | | 0.73143884 | |  |
| Liberia | 0.01099166 | | 0.19661659 | | 0.09604736 | | 0.30991509 | |  |
| Libya | 0.89627005 | | 0.39680407 | | 0.04523701 | | -0.2126899 | |  |
| Lithuania | 0.30306157 | | 0.39392987 | | 0.03656998 | | -0.1912328 | |  |
| Luxembourg | 2.2164E-06 | | 0.08072062* | | 0.1216782 | | -0.348824 | |  |
| Madagascar | 8.9467E-09 | | 0.27576994 | | 0.06215798 | | -0.249315 | |  |
| Malawi | 3.4824E-06 | | 0.05453651 | | 0.18096814 | | 0.42540351 | |  |
| Malaysia | 6.2209E-05 | | 0.00067204*** | | 0.3883476 | | -0.6231754 | |  |
| Maldives | 0.00021655 | | 0.07073804* | | 0.15410734 | | -0.3925651 | |  |
| Mali | 1.3967E-07 | | 0.8890662 | | 0.00099693 | | -0.0315742 | |  |
| Malta | 0.13537534 | | 0.00039846*** | | 0.45705222 | | -0.6760564 | |  |
| Marshall Islands | 0.95507312 | | 0.20716416 | | 0.08685931 | | 0.29471903 | |  |
| Mauritania | 1.5288E-11 | | 0.22866134 | | 0.07530482 | | 0.27441723 | |  |
| Mauritius | 0.17120572 | | 0.03968796** | | 0.19491863 | | -0.4414959 | |  |
| Mexico | 5.5552E-07 | | 2.937E-05*** | | 0.50912999 | | -0.7135335 | |  |
| Moldova | 0.72767763 | | 0.67812527 | | 0.00797707 | | 0.08931443 | |  |
| Mongolia | 5.985E-10 | | 2.2673E-05*** | | 0.66192435 | | -0.8135873 | |  |
| Montenegro | 0.00490813 | | 0.0030067** | | 0.53360009 | | -0.7304794 | |  |
| Morocco | 4.1945E-15 | | 5.7776E-08*** | | 0.76103345 | | -0.8723723 | |  |
| Mozambique | 7.2247E-09 | | 5.1335E-08*** | | 0.78041716 | | 0.88341223 | |  |
| Myanmar | 0.20015413 | | 0.76733792 | | 0.00562834 | | -0.0750223 | |  |
| Namibia | 0.82541578 | | 0.01542662** | | 0.24869856 | | 0.49869686 | |  |
| Nepal | 2.9537E-08 | | 0.00673118*** | | 0.3008191 | | -0.5484698 | |  |
| Netherlands | 0.103288 | | 0.19385636 | | 0.06655962 | | -0.2579915 | |  |
| New Zealand | 0.00361646 | | 0.00864828** | | 0.25408662 | | -0.5040701 | |  |
| Nicaragua | 3.0051E-07 | | 0.01029136** | | 0.26358214 | | -0.5134025 | |  |
| Niger | 3.8382E-17 | | 0.4854087 | | 0.02464016 | | 0.15697185 | |  |
| Nigeria | 5.7369E-06 | | 0.00207355** | | 0.38470914 | | 0.62024925 | |  |
| North America | 0.00855926 | | 0.00502698** | | 0.27455571 | | -0.5239806 | |  |
| North Macedonia | 0.15149718 | | 0.1865781 | | 0.08153763 | | -0.285548 | |  |
| Northern Mariana Islands | | 0.1514971 | | 0.1865781 | | 0.0815376 | | -0.285548 | |
| Norway | 0.08730215 | | 0.54346827 | | 0.01495091 | | 0.12227392 | |  |
| Oman | 4.1406E-05 | | 0.02892216** | | 0.22718516 | | -0.4766394 | |  |
| Pakistan | 0.00638245 | | 0.25526051 | | 0.06418944 | | 0.25335635 | |  |
| Panama | 0.05318971 | | 0.7063701 | | 0.00657779 | | -0.0811036 | |  |
| Papua New Guinea | 0.42994004 | | 0.14195214 | | 0.10464595 | | 0.32349026 | |  |
| Paraguay | 0.00910802 | | 0.72919673 | | 0.00612524 | | 0.07826394 | |  |
| Peru | 2.1113E-09 | | 4.442E-05*** | | 0.53888349 | | -0.7340868 | |  |
| Philippines | 0.01041116 | | 0.96657981 | | 8.1624E-05 | | -0.0090346 | |  |
| Poland | 0.00111279 | | 0.01345313** | | 0.22055281 | | -0.4696305 | |  |
| Portugal | 0.01515995 | | 0.0878758 | | 0.11205335 | | -0.3347437 | |  |
| Puerto Rico | 0.10295272 | | 0.04950423** | | 0.17144818 | | -0.414063 | |  |
| Qatar | 8.6241E-09 | | 0.01095842** | | 0.27051342 | | -0.520109 | |  |
| Romania | 0.22467571 | | 0.40933924 | | 0.02979222 | | -0.1726042 | |  |
| Rwanda | 3.6188E-07 | | 0.07650305 | | 0.15589417 | | -0.3948344 | |  |
| Samoa | 0.87846904 | | 0.97626403 | | 4.7832E-05 | | -0.0069161 | |  |
| Sao Tome and Principe | 7.8794E-05 | | 0.74697585 | | 0.00592784 | | -0.0769925 | |  |
| Saudi Arabia | 1.8458E-13 | | 2.7783E-07*** | | 0.7230818 | | -0.8503422 | |  |
| Senegal | 5.1296E-12 | | 0.6372459 | | 0.0107849 | | 0.10385037 | |  |
| Serbia | 0.60628563 | | 0.4316043 | | 0.05230432 | | 0.22870138 | |  |
| Seychelles | 0.00031525 | | 0.00142752** | | 0.42238596 | | -0.6499123 | |  |
| Sierra Leone | 0.00020735 | | 0.2289195 | | 0.07151204 | | 0.26741735 | |  |
| Singapore | 1.4654E-07 | | 2.4352E-05*** | | 0.51619895 | | -0.7184699 | |  |
| Slovenia | 0.18262508 | | 0.38303709 | | 0.03324361 | | -0.1823283 | |  |
| Solomon Islands | 0.0053714 | | 0.18117663 | | 0.08348382 | | 0.28893566 | |  |
| Somalia | 0.00171583 | | 0.41264996 | | 0.03561275 | | 0.1887134 | |  |
| South Africa | 0.01065111 | | 9.2894E-06*** | | 0.55106785 | | 0.74233945 | |  |
| South Asia | 3.2209E-12 | | 0.41677156 | | 0.02766207 | | -0.1663192 | |  |
| South Sudan | 0.4984329 | | 0.38521036 | | 0.2552896 | | 0.50526191 | |  |
| Spain | 0.44204631 | | 0.65366092 | | 0.00818149 | | 0.0904516 | |  |
| Sri Lanka | 0.77644771 | | 0.88736336 | | 0.00093244 | | 0.03053587 | |  |
| Sub-Saharan Africa | 0.00118249 | | 0.00760661** | | 0.25215894 | | 0.5021543 | |  |
| Sudan | 3.5708E-05 | | 0.06989043 | | 0.19082784 | | 0.43683846 | |  |
| Suriname | 3.851E-05 | | 0.00896272** | | 0.28312876 | | -0.5320984 | |  |
| Sweden | 0.00047018 | | 0.15011904 | | 0.0810307 | | -0.2846589 | |  |
| Switzerland | 0.00446003 | | 0.22531505 | | 0.05822542 | | -0.2412994 | |  |
| Tajikistan | 1.6988E-06 | | 0.00023675*** | | 0.55824738 | | -0.7471595 | |  |
| Tanzania | 3.0859E-10 | | 0.01043021** | | 0.28541572 | | 0.53424313 | |  |
| Thailand | 0.15977729 | | 0.71431409 | | 0.00545352 | | -0.073848 | |  |
| Togo | 0.00112446 | | 6.167E-06*** | | 0.64842582 | | 0.80524892 | |  |
| Tonga | 0.87911481 | | 0.67239978 | | 0.00868168 | | 0.09317555 | |  |
| Trinidad and Tobago | 0.19851567 | | 0.26257121 | | 0.05936587 | | -0.2436511 | |  |
| Tunisia | 1.756E-10 | | 6.4687E-07*** | | 0.68308886 | | -0.8264919 | |  |
| Turkey | 2.2882E-06 | | 0.00369181** | | 0.3122251 | | -0.5587711 | |  |
| Turkmenistan | 0.00045435 | | 0.16128974 | | 0.11203651 | | -0.3347186 | |  |
| Uganda | 1.3921E-11 | | 0.00835306** | | 0.28751172 | | 0.5362012 | |  |
| Ukraine | 0.92562969 | | 0.92892999 | | 0.00035341 | | -0.0187992 | |  |
| United Arab Emirates | 0.00646428 | | 0.00377353** | | 0.33554885 | | -0.5792658 | |  |
| United Kingdom | 0.00024557 | | 0.02594715** | | 0.18316527 | | -0.4279781 | |  |
| United States | 0.00993097 | | 0.00655042** | | 0.26029882 | | -0.5101949 | |  |
| Uruguay | 0.00397285 | | 0.10306183 | | 0.11621575 | | -0.3409043 | |  |
| Uzbekistan | 6.2351E-07 | | 0.14733808 | | 0.09730237 | | -0.3119333 | |  |
| Vanuatu | 0.76007126 | | 0.95475372 | | 0.00016502 | | 0.01284604 | |  |
| Vietnam | 3.7444E-08 | | 3.3087E-05** | | 0.58593891 | | -0.7654665 | |  |
| World | 5.6397E-11 | | 0.60279281 | | 0.01098989 | | -0.1048327 | |  |
| Zambia | 0.0016289 | | 0.00651346** | | 0.29105625 | | 0.53949629 | |  |
| Zimbabwe | 0.17029572 | | 0.0001509*** | | 0.48671018 | | 0.69764617 | |  |

Summary table 173 countries, DALY mental health condition of population regressed on internet usage per country population from 1990-2019. Signif. codes: 0 ‘***’ 0.001’**’ 0.05’*’ 0.10’.‘ 1.
